# Serum Leptin Exacerbates Osteoarthritis by Promoting Subchondral Bone H‐Type Vessel Angiogenesis via Activation of the PI3K/AKT Pathway

**DOI:** 10.1111/jcmm.71232

**Published:** 2026-06-29

**Authors:** Ruifu Li, Zhao Xi, Shitong Luo, Jun Qin, Tao Liu, Jian Zhang

**Affiliations:** ^1^ University Town Hospital of Chongqing Medical University Chongqing China; ^2^ The First Affiliated Hospital of Chongqing Medical University Chongqing China

**Keywords:** H‐type vessels, leptin, osteoarthritis, PI3K‐AKT Signalling pathway, subchondral bone

## Abstract

Osteoarthritis (OA) is a common degenerative joint disease closely associated with obesity. Adipokines secreted by adipose tissue play critical roles in disease progression. This study focuses on the adipokine leptin and investigates its relationship with knee osteoarthritis. Analysis of subchondral bone tissue from OA patients revealed decreased leptin receptor expression, enhanced osteogenic activity, and a significant increase in H‐type vessel number. Concurrently, serum leptin levels were markedly elevated in OA patients. Animal experiments further demonstrated that leptin‐knockout obese mice exhibited significantly milder OA, reduced H‐type vessel formation, and decreased osteogenic activity. In contrast, mice with high serum leptin levels showed more severe joint destruction and vascular hyperplasia. In vitro cell experiments indicated that leptin activates the PI3K/AKT signalling pathway in bone marrow mesenchymal stem cells (BMSCs), increasing the secretion of angiogenic factors such as VEGF and HIF‐1α. Conditioned medium from leptin‐treated BMSCs promoted tube formation and expression of H‐type vessel markers (EMCN/CD31) in mouse umbilical vein endothelial cells. Inhibition of the PI3K pathway using Ly294002 in BMSCs or ZSTK474 in mice abolished leptin's effects. These results suggest that obesity‐induced hyperleptinemia drives aberrant H‐type vessel angiogenesis and active bone remodelling in subchondral bone via the PI3K/AKT pathway, thereby accelerating OA progression.

## 1 Introduction

Osteoarthritis (OA) is the most common degenerative joint disease and represents the most prevalent degenerative disorder among the elderly population. Recent global epidemiological studies on OA indicate that approximately 240 million people worldwide suffer from symptomatic OA. The majority of these patients are over 60 years of age, and the annual incidence rate is increasing by 181 per 100,000 people each year [1]. The pathogenesis of OA is not fully understood [2]. However, current theory suggests that OA is not a single disease caused by a sole pathogenic factor, but rather a constellation of pathological changes resulting from the accumulation of a series of risk factors. Obesity and its associated metabolic syndrome (Mets) are among the most frequent acquired risk factors and are believed to play significant roles in the initiation and progression of OA [3, 4].

In obese patients, a high BMI leads to increased mechanical stress on the joints. Concurrently, decreased muscle strength elevates the probability of joint injury and repetitive microtrauma, both of which promote the development of OA [4, 5]. Furthermore, adipose tissue serves not only as an energy storage organ but also as an important immune organ. White adipose tissue can release a series of pro‐inflammatory factors, including IL‐1, TNF‐α, and IL‐6. These pro‐inflammatory factors can induce degradation of the cartilage matrix, promote chondrocyte apoptosis, and stimulate osteophyte formation through pathways such as activating matrix metalloproteinases (MMPs), inducing nitric oxide synthase (iNOS), and promoting M1 macrophage polarization and infiltration, thereby exacerbating OA [6, 7, 8, 9, 10, 11, 12].

In addition to inflammatory cytokines, recent studies have revealed that adipokines secreted by adipose tissue also play crucial roles in the pathogenesis of OA. Certain adipokines, such as Adiponectin (APN), Vaspin, and Omentin, are considered to have anti‐inflammatory and cartilage‐protective effects [13]. Conversely, adipokines with pro‐inflammatory and joint‐destructive properties include Leptin, Resistin, and Visfatin [14, 15, 16, 17]. Among these, Leptin is the most classical and extensively studied adipokine. Clinical studies have shown that serum and synovial fluid leptin levels are significantly elevated in obese individuals and OA patients, exhibiting a positive correlation with both BMI and OA severity [18, 19]. As a pro‐inflammatory factor, leptin can induce immune‐mediated damage to joints by promoting the production of IL‐1, IL‐6, IL‐12, and TNF‐α [20, 21]. Leptin can also induce chondrocyte apoptosis via activation of the JAK2/STAT3 and JNK signalling pathways [22, 23, 24]. Furthermore, leptin can induce cartilage matrix degradation and promote osteophyte formation [9].

Nevertheless, the relationship between leptin and osteoarthritis remains incompletely elucidated, with many conclusions still relying on in vitro data. The correlations between leptin levels in serum, intra‐articular adipose tissue, and articular cartilage and OA also remain contentious. Moreover, most studies have focused on the articular cartilage and intra‐articular environment, with relatively few investigating the relationship between serum leptin and changes in the subchondral bone. The aim of this study is to explore the relationship between serum leptin levels and alterations in the subchondral bone, and its correlation with OA, through clinical specimen research and in vitro and in vivo experiments.

## 2 Materials and Methods

### 2.1 Experimental Animals and Housing

Seven‐week‐old male C57BLKS/J (BKS) mice and leptin gene‐knockout C57BLKS‐Lep^ob^/J (ob/ob) mice were purchased from GEM pharmatech Biosciences Inc. (Chengdu, China). The mice were housed in the Animal Experiment Center of Jinyun Campus, Chongqing Medical University, under specific pathogen‐free (SPF) conditions: temperature 22°C ± 2°C, humidity 55% ± 5%, with a 12‐h light/dark cycle. They were fed a high‐fat diet (MD12033, Medicience, China) with free access to food and water.

### 2.2 Osteoarthritis Modelling and Drug Administration

At 8 weeks of age, all mice underwent destabilization of the medial meniscus (DMM) surgery on the right knee to induce OA. Briefly, the anterior attachment of the medial meniscus to the tibial plateau was transected to destabilize the knee joint. Four days post‐surgery, after confirming wound healing and suture loss, gradual running wheel training commenced at a speed of 10 m/min for a distance of 600 m/day.

For the ZSTK474 intervention group (BKS + ZSTK474), ZSTK474 (Cat# SC0411, Beyotime, China) was dissolved in DMSO (20 mg/mL) and then diluted in 0.5% sodium carboxymethyl cellulose (CMC‐Na) solution to a final concentration of 6 mg/mL. It was administered daily via oral gavage at a dose of 400 mg/kg/day, starting from the day of surgery. Mice were euthanized at 8 weeks post‐surgery (16 weeks of age), and cardiac blood along with bilateral knee joint specimens were collected.

### 2.3 Cell Culture

#### 2.3.1 Isolation of BMSCs

Primary mouse bone marrow mesenchymal stem cells (BMSCs) were isolated from the bone marrow of femurs and tibiae of C57BL/6J mice using a separation kit (IMP‐MK013, IMMOCELL, China). The cells were seeded in DMEM/F‐12 low‐glucose medium (C8015, Adamas Life, China) supplemented with 10% premium fetal bovine serum (C8090, Adamas Life, China) and cultured at 37°C with 5% CO_2_.

#### 2.3.2 Leptin Treatment

After trypsinization, BMSCs were seeded into 6‐well plates. When cell confluence reached approximately 65%, recombinant mouse leptin protein (TP02803, TZYBIO, China), reconstituted in PBS, was added to the culture medium to achieve final concentrations of 500 ng/mL or 1000 ng/mL, according to the experimental group sets. After 24 h of treatment (when confluence reached ~90%), subsequent experiments were performed.

#### 2.3.3 Preparation of Conditioned Medium (CM)

The aforementioned leptin‐treated (1000 ng/mL) and untreated BMSCs were washed three times with PBS and then cultured in serum‐free DMEM/F‐12 low‐glucose medium for 24 h. The culture supernatant was collected, filtered through a 40‐μm cell strainer, and used as conditioned medium.

#### 2.3.4 Culture of MUVECs

Immortalized mouse umbilical vein endothelial cells (MUVECs, HTX3109, Otwobio, China) were cultured in endothelial cell complete medium (G4567, Servicebio, China) containing 10% premium fetal bovine serum at 37°C with 5% CO_2_.

### 2.4 RNA Extraction and RT‐PCR Analysis

Tissue specimens were snap‐frozen in liquid nitrogen and ground using a mortar and pestle. Both cells and tissue fragments were homogenized using a bead mill. Total RNA was extracted using Beyozol reagent (R0011, Beyotime, China) according to the manufacturer's instructions. cDNA was synthesized from total RNA using the BeyoRT III First Strand cDNA Synthesis Kit (D7178M, Beyotime, China) and stored at −20°C.

The primer sequences used for RT‐PCR were as follows:

*Human RUNX2*: Forward: 5′‐CGCCTCACAAACAACCACAG‐3′; Reverse: 5′‐TCACTGTGCTGAAGAGGCTG‐3′.

*Mouse RUNX2*: Forward: 5′‐CGCCTCACAAACAACCACAG‐3′; Reverse: 5′‐TCACTGTGCTGAAGAGGCTG‐3′.

*Human Leptin*: Forward: 5′‐CCCCCTGCTCTTTGTTTCCT‐3′; Reverse: 5′‐AGTGGATCCCTTCTTCCCGA‐3′.

*Mouse Leptin*: Forward: 5′‐AAGGGGCTTGGGTTTTTCCA‐3′; Reverse: 5′‐TGCCCTGAAATGCGGTATGT‐3′.

*Human Leptin Receptor (LEPR)*: Forward: 5′‐ACAGCATCAGTGACATGTGGT‐3′; Reverse: 5′‐TCCGTGAATAAACAGGGGGC‐3′.

*Mouse Leptin Receptor (LEPR)*: Forward: 5′‐CTCGGGGTTGGATGAGCTTT‐3′; Reverse: 5′‐CTGTGCGTGGAACAGGTTTG‐3′.

*Human OPN*: Forward: 5′‐ATCTCCTAGCCCCACAGACC‐3′; Reverse: 5′‐GTGGGTTTCAGCACTCTGGT‐3′.

*Mouse OPN*: Forward: 5′‐CCTTGCTTGGGTTTGCAGTC‐3′; Reverse: 5′‐ACAGGGATGACATCGAGGGA‐3′.

*Mouse OCN*: Forward: 5′‐AGACAAGTCCCACACAGCAG‐3′; Reverse: 5′‐GGGCAGCACAGGTCCTAAAT‐3′.

RT‐PCR was performed on a LightCycler 96 System (Roche, Switzerland) using a Real‐Time Fluorescent Quantitative PCR Kit (D7262, Beyotime, China). The thermocycling conditions were: 95°C for 2 min (pre‐denaturation), followed by 40 cycles of 95°C for 15 s and 60°C for 15–30 s. The relative gene expression levels were calculated using the 2^−ΔΔCt^ method with the instrument's built‐in analysis software.

### 2.5 Enzyme‐Linked Immunosorbent Assay (ELISA)

Serum leptin levels were measured using a species‐specific leptin ELISA kit (XINYU Biotech, Shanghai, China) according to the manufacturer's instructions. Samples were diluted 1:5 with sample diluent. Then, 100 μL of HRP‐conjugated detection antibody was added to each well. Plates were incubated at 37°C for 60 min. After incubation, wells were aspirated and washed five times with wash buffer. Subsequently, 50 μL of Substrate A and 50 μL of Substrate B were added to each well, followed by incubation at 37°C in the dark for 15 min. The reaction was stopped by adding 50 μL of stop solution. The optical density (OD) at 450 nm was measured within 15 min using a microplate reader (Infinite 200, TECAN). Sample concentrations were calculated based on the standard curve.

### 2.6 Western Blot Analysis

Tissue samples were snap‐frozen, ground under liquid nitrogen, and further disrupted using a low‐temperature bead mill. Total protein was extracted using RIPA lysis buffer (P0013B, Beyotime, China) supplemented with 1% universal protease inhibitor cocktail (P1005, Beyotime, China), following the manufacturer's protocol. For cell samples, the lysis buffer mixture was added directly to the culture dish for digestion. Protein concentration was determined using a BCA assay kit (P0012S, Beyotime, China).

Electrophoresis was performed using the MOPS‐SDS Running Buffer system on 4%–12% gradient polyacrylamide gels (ET12012Gel, ACE, China). A total of 25 μg of protein per sample was loaded into each well. After electrophoresis, proteins were transferred onto a polyvinylidene fluoride (PVDF) membrane (Millipore) via wet transfer. Following blocking, the membrane was incubated with primary antibody at 5°C overnight. After washing, the membrane was incubated with an HRP‐conjugated secondary antibody. Protein bands were visualized using an ECL ultrasensitive chemiluminescence kit (BeyoECL Plus, Beyotime, China) and imaged with a chemiluminescence imaging system.

### 2.7 Histology and Immunostaining

Human and mouse knee joint specimens were used for Safranin O/Fast Green staining, immunohistochemistry (IHC), and immunofluorescence (IF) staining. Tissues were fixed in 4% paraformaldehyde for 24 h, followed by decalcification in 10% EDTA solution for 8 weeks (with weekly solution changes). After decalcification, tissues were dehydrated through an ethanol gradient, embedded in paraffin, and sectioned into 4‐μm thick slices using a microtome.

#### 2.7.1 Safranin O/Fast Green Staining

Sections were deparaffinized and rehydrated. They were stained with 1% Safranin O solution overnight, differentiated in 1% acid alcohol for 30 s, rinsed, dehydrated through an ethanol series, and counterstained with 0.5% Fast Green solution for 45 s. Following final dehydration and clearing in xylene, sections were mounted with neutral resin.

#### 2.7.2 Immunohistochemistry (IHC)

After deparaffinization, rehydration, and antigen retrieval using Tris‐EDTA buffer (G1203, Servicebio, China), endogenous peroxidase activity was blocked with 3% H_2_O_2_. Sections were blocked with 3% BSA and incubated with primary antibody at 4°C overnight. After washing, HRP‐labelled secondary antibody was applied for 50 min. Colour development was performed using DAB substrate, monitored under a microscope, and stopped by rinsing. Sections were counterstained with haematoxylin, differentiated, blued, dehydrated, cleared, and mounted.

#### 2.7.3 Immunofluorescence Double Staining (IF)

Deparaffinized and rehydrated sections underwent antigen retrieval and peroxidase blocking. After blocking with 3% BSA, the first primary antibody was applied overnight at 4°C. After washing, the corresponding fluorophore‐conjugated secondary antibody was added for 50 min at room temperature. Signal amplification was achieved using a TSA kit (iF488‐Tyramide, G1231, servicebio, China). Antigens were then retrieved again using the appropriate buffer via microwave heating. Subsequently, the second primary antibody was applied overnight at 4°C, followed by the corresponding secondary antibody (incubated for 50 min at RT) and another TSA dye (CY3‐Tyramide, G1223, servicebio, China). Nuclei were counterstained with DAPI. Autofluorescence was quenched using quenching reagent B for 5 min. Sections were mounted with anti‐fade mounting medium and imaged using a fluorescence microscope.

### 2.8 Tubulogenesis Assay

Cryopreserved MUVECs were thawed and cultured for 4 days. Cells were trypsinized, resuspended, and seeded into 6‐well plates. After 24 h, the medium was replaced with serum‐free medium for starvation for 6 h. Cells were then treated according to the experimental groups set for 24 h. Low‐growth‐factor Matrigel (Matrigel Matrix, Corning, 354,230, USA) was thawed overnight at 4°C. Using pre‐cooled tips and plates kept on ice, 60 μL of Matrigel was added to each well of a 96‐well plate and allowed to polymerize at 37°C for 30 min. MUVECs were trypsinized, and a cell suspension of 2 × 10^5^ cells/ml was prepared. Then, 100 μL of the cell suspension was added to each Matrigel‐coated well. The plate was incubated at 37°C with 5% CO_2_. Tube formation was observed and photographed under an inverted microscope every 2 h. Images were analysed using ImageJ software to quantify total tube length and length of branches.

### 2.9 Micro‐CT Analysis

After euthanasia, mouse knee joints were harvested and fixed in 4% paraformaldehyde for 24 h. After rinsing with PBS, the knees were fixed in a 15° flexed position on sponge holders and placed in scanning tubes. Scanning was performed at a resolution of 5 μm, with a voltage of 70 kV and an exposure time of 200 ms. After scanning, parameters such as osteophyte number, bone volume fraction (BV/TV), trabecular thickness (Tb.Th), trabecular separation (Tb.Sp), trabecular number (Tb.N), subchondral bone plate thickness, and bone mineral density (BMD) were analysed using CTAn software (version 1.18, Bruker, USA).

### 2.10 Statistical Analysis

Data are expressed as mean ± standard error of the mean (SEM). Statistical analysis was performed using one‐way analysis of variance (ANOVA). All experiments were repeated independently at least three times. A *p*‐value of < 0.05 was considered statistically significant.

## 3 Results

### 3.1 Bioinformatic Analysis Reveals Decreased LEP Expression in the Subchondral Bone of Osteoarthritis Patients

To identify potential factors contributing to OA, we initially performed bioinformatic analysis. The whole‐genome expression profiles were obtained from the GEO (Gene Expression Omnibus) database, dataset GSE51588. This dataset contained genome‐wide expression profiling of total RNA extracted from the medial and lateral tibial plateau subchondral bone of 20 OA patients. Reverse verification via qRT‐PCR was performed for 85 differentially expressed genes, confirming the dataset's high reliability.

**FIGURE 1:**
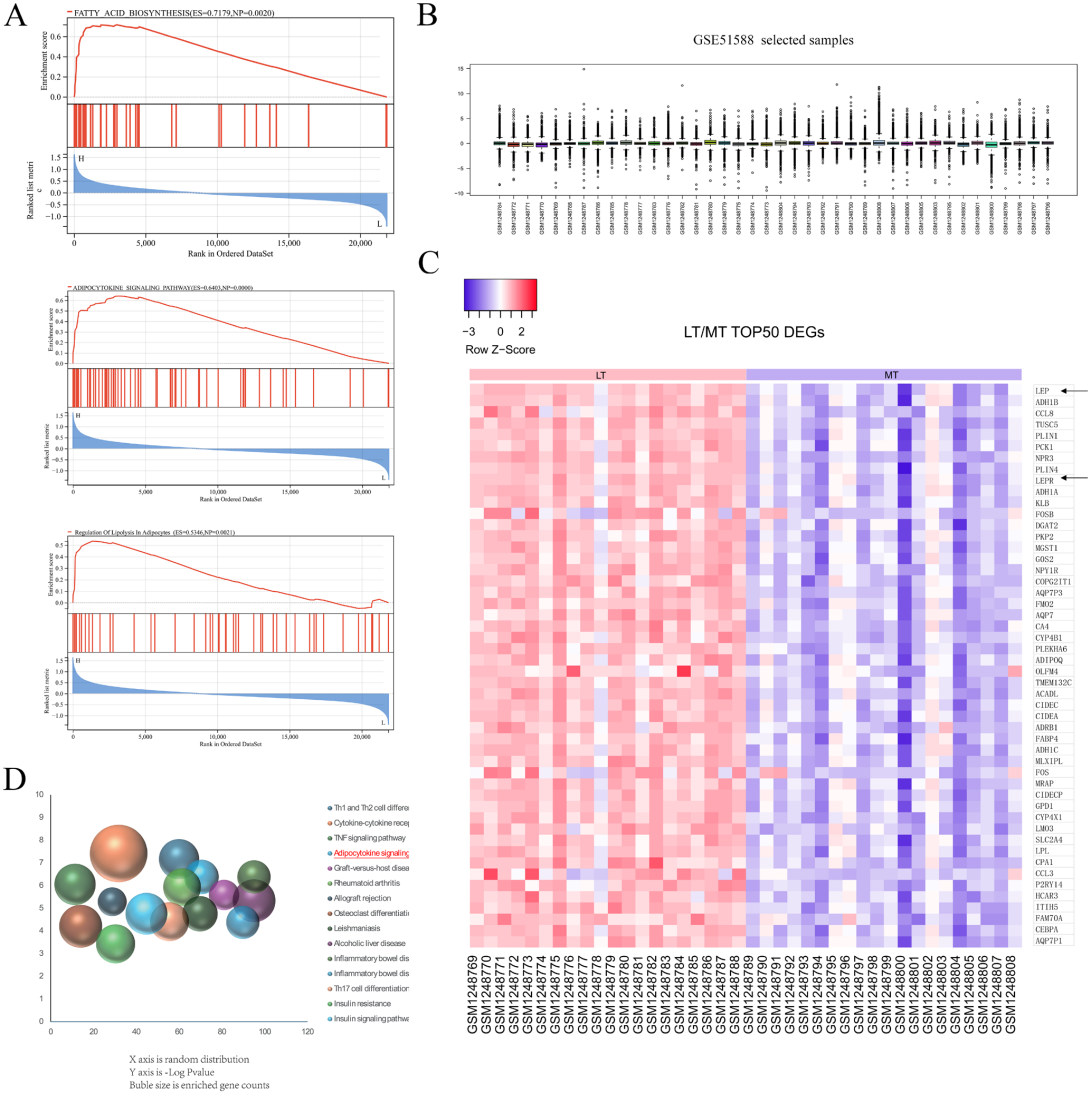
(A) Results of gene set enrichment analysis for differentially expressed genes. (B) Data sourced from the GEO dataset GSE51588. The basic distribution of differentially expressed genes from an unpaired analysis of the medial and lateral tibial plateau subchondral bone of 20 OA patients is shown. (C) Hotspot map analysis results of the differentially expressed genes. (D) KEGG pathway enrichment analysis of differentially expressed genes.

Gene set enrichment analysis of the differentially expressed genes revealed significant enrichment in the following pathways: Fatty Acid Biosynthesis, Adipocytokine Signalling Pathway, Regulation of Lipolysis in Adipocytes, and the Insulin Signalling Pathway (Figure 1A). Network analysis indicated complex interconnections among these four pathways. This suggests significant expression differences at the level of lipid metabolism and adipokine‐related pathways between the damaged medial and the non‐damaged lateral tibial plateau subchondral bone. Furthermore, Protein–Protein Interaction (PPI) analysis of the differentially expressed genes identified Leptin (LEP) as a potential hub gene (Figure 1C). As a crucial adipokine extensively involved in lipid metabolism and the regulation of insulin function, it is reasonable to hypothesize that LEP likely serves as a key regulator of the aforementioned four enriched pathways.

### 3.2 Elevated Serum Leptin Levels in OA Patients Are Associated With Reduced LEP/LEPR Expression in Subchondral Bone, Increased Osteogenic Activity, and Abundant H‐Type Vessels

**FIGURE 2:**
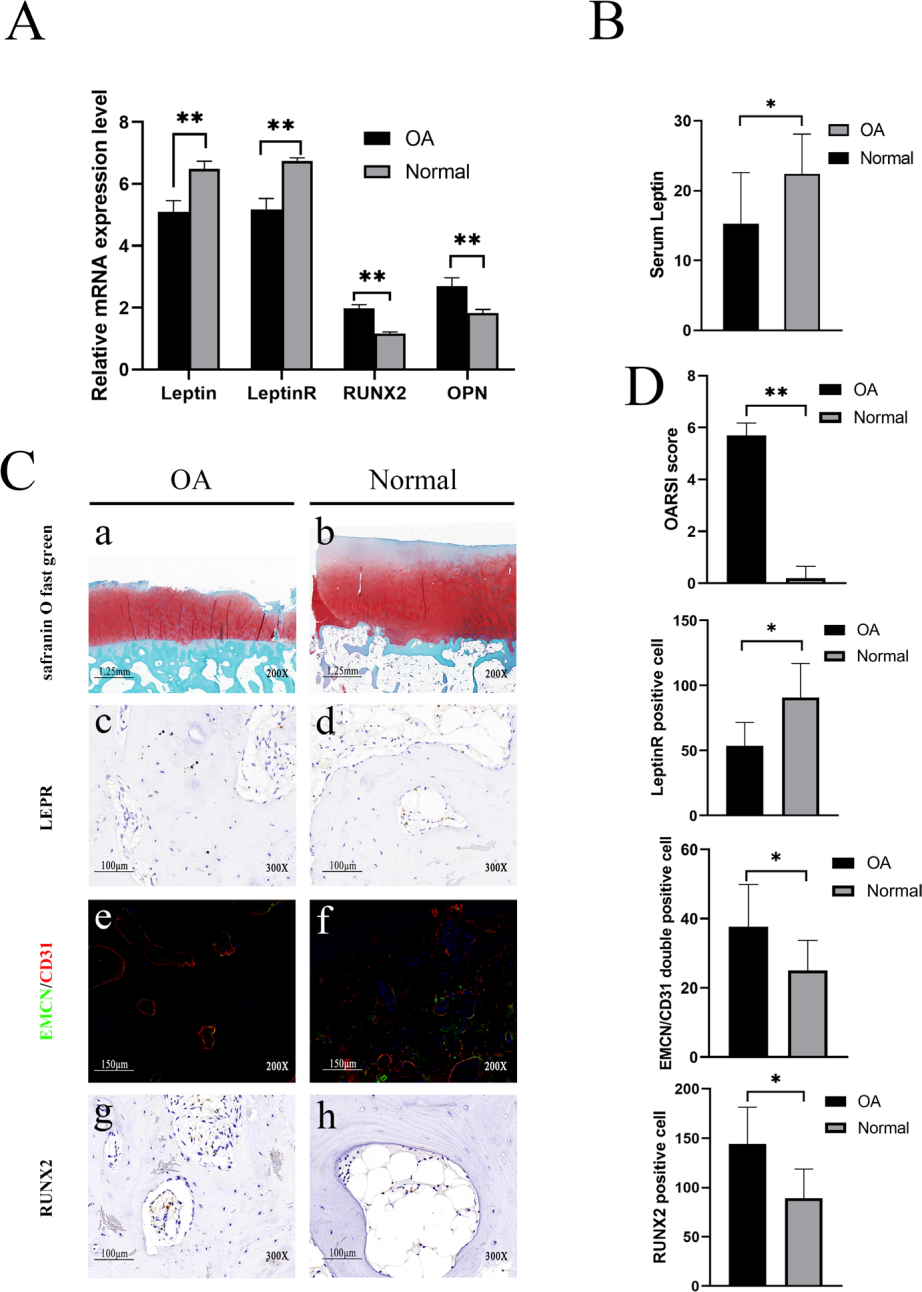
(A) Relative expression levels of LEP, LEPR, RUNX2, and OPN in total RNA extracted from the subchondral bone of OA patients and healthy controls, detected by RT‐PCR. Sample size: *N* = 10 for OA patients, *n* = 5 for healthy controls. Statistical analysis was performed using Welch's corrected *t*‐test. (B) Serum leptin concentration in peripheral blood of OA patients and healthy controls measured by ELISA. Sample size: *N* = 10 per group. An independent samples *t*‐test was used. (C, D) Histological analysis of the knee joint subchondral bone from OA patients and healthy controls, including OARSI scoring, LEPR and RUNX2 immunohistochemical staining, and immunofluorescence double staining for H‐type vessel‐specific markers. Sample size: *N* = 10 for OA patients, *n* = 5 for healthy controls. Statistical analysis was performed using Welch's corrected *t*‐test. *p < 0.05, **p < 0.01.

To validate this hypothesis, we extracted total RNA from the subchondral bone of the tibial plateau from 10 OA patients and 5 non‐OA patients (who underwent amputation due to trauma). RT‐PCR analysis revealed significantly decreased expression of both LEP and its receptor LEPR in the subchondral bone of OA patients. Conversely, the expression of osteogenic marker genes RUNX2 and OPN was markedly increased (Figure 2A).

Subsequently, we collected peripheral blood from 10 OA patients and 10 non‐OA individuals. ELISA results demonstrated that the serum leptin concentration was significantly higher in OA patients compared to the non‐OA group (Figure 2B).

Immunohistochemical staining further showed a significant reduction in the number of LEPR‐positive cells in the tibial plateau subchondral bone of OA patients. In parallel, the number of cells positive for the osteogenic markers RUNX2 and OPN was increased. Immunofluorescence double staining for the H‐type vessel‐specific markers EMCN and CD31 indicated a substantial increase in the number of H‐type vessels within the subchondral bone of OA patients compared to non‐OA individuals (Figure 2C,D).

In summary, OA patients generally exhibit higher serum leptin levels. However, in their subchondral bone, LEPR expression is decreased, osteogenic activity is elevated, and H‐type vessel numbers are increased. Does this suggest that high serum leptin levels contribute to H‐type vessel hyperplasia and increased osteogenic activity in the knee joint, thereby promoting the development of OA?

### 3.3 Genetic Knockout of Leptin Attenuates OA Severity and Reduces H‐Type Vessel Formation in Obese Mice

**FIGURE 3:**
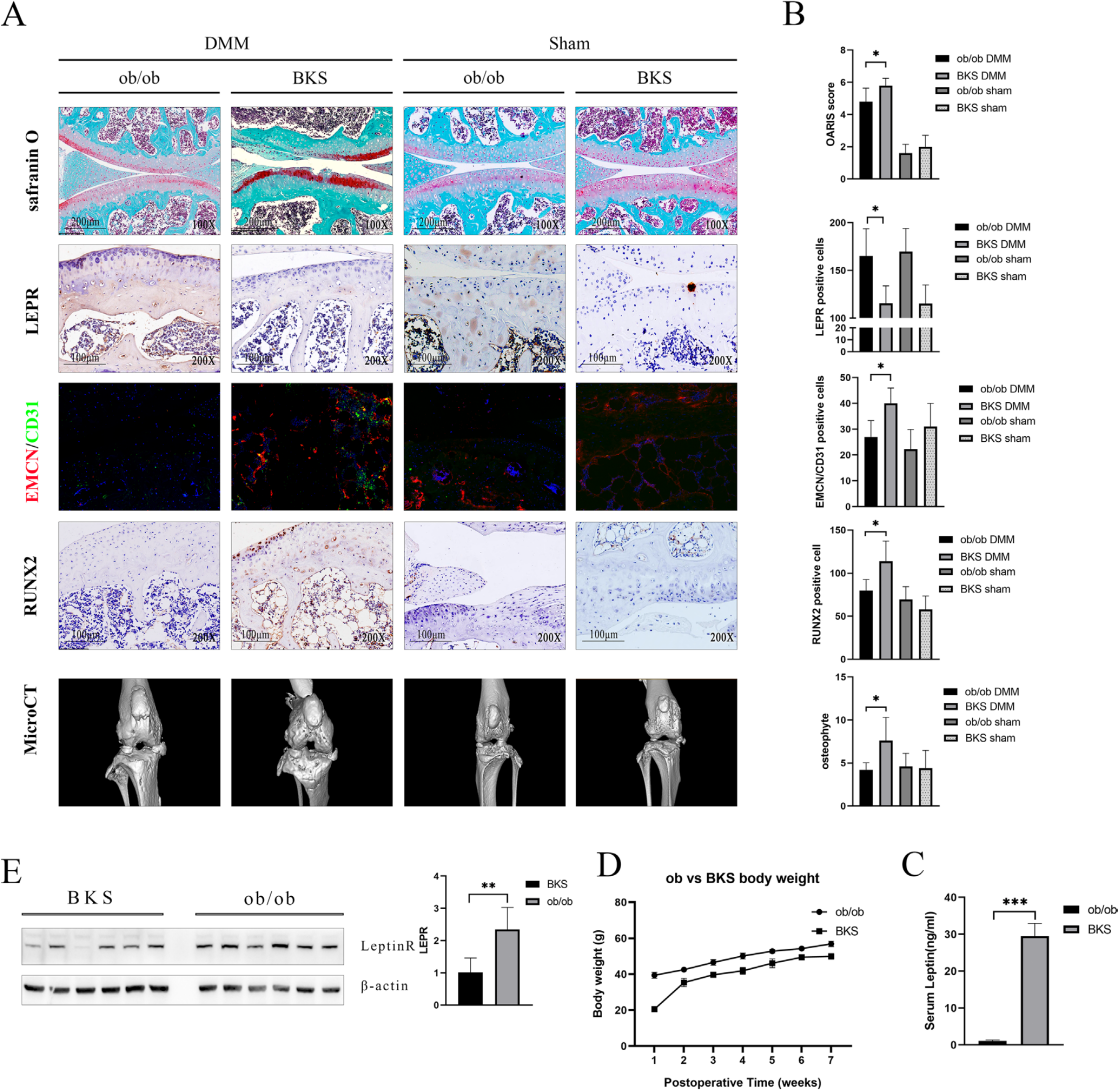
(A, B) Histological images and quantitative results of mouse knee joints 7 weeks after DMM surgery and sham operation. From top to bottom: Safranin O/Fast Green staining, LEPR immunohistochemical staining, immunofluorescence double staining for H‐type vessel markers, RUNX2 immunohistochemical staining, and Micro‐CT quantification of osteophyte number (*n* = 5 per group). (C) Serum leptin concentration in mice (*n* = 5). (D) Body weight change curve of mice after DMM surgery. (E) The expression level of LEPR in total protein extracts from the subchondral bone (*n* = 5). All data are presented as mean ± SEM. *p < 0.05, **p < 0.01, ***p < 0.001.

To validate the hypothesis that serum leptin influences OA progression, we established an animal model. Five male C57BLKS‐Lep^ob^/J (ob/ob) mice with a knockout of the LEP gene and five male wild‐type C57BLKS/J (BKS) mice were selected. At 8 weeks of age, destabilization of the medial meniscus (DMM) surgery was performed on the right knee of all mice in both groups to induce osteoarthritis; the left knee underwent a sham operation as a control. Postoperatively, mice were fed a high‐fat diet. They underwent daily running wheel exercise at 10 m/min for 60 min, and body weight was measured weekly (Figure 3D). At 15 weeks, mice were euthanized, blood was collected for serum leptin measurement via ELISA, and knee joint specimens were harvested for micro‐CT scanning. Subsequently, joints were fixed, decalcified, and sectioned.

Safranin O/Fast Green staining revealed more severe cartilage erosion and wear in the BKS group compared to the ob/ob group, accompanied by a significantly higher OARSI score. Micro‐CT analysis showed a markedly greater number of osteophytes in the BKS group compared to the ob/ob group. Immunohistochemical staining indicated a significantly higher number of LEPR‐positive cells in the tibial plateau subchondral bone of ob/ob mice compared to the BKS group, while the number of RUNX2‐positive cells was significantly lower in the ob/ob group (Figure 3A). Western blot analysis of total protein extracted from homogenized knee joint subchondral bone showed significantly higher LEPR levels in the ob/ob group compared to the BKS group (Figure 3E). These results demonstrate that mice with low serum leptin levels exhibit less severe OA. Concurrently, the content of LEPR in their subchondral bone was compensatorily increased. In contrast, mice with high serum leptin showed increased H‐type vessel numbers and elevated RUNX2 expression in the subchondral bone.

These findings suggest that high serum leptin levels are associated with exacerbated OA pathology, increased osteogenic activity, and H‐type vessel formation in the subchondral bone. Consequently, this raises the question: Does serum leptin influence OA pathogenesis primarily by affecting H‐type vessel differentiation, or does it also act directly on the subchondral bone?

### 3.4 Leptin Does Not Directly Affect BMSC Differentiation or MUVEC Morphogenesis, but Conditioned Medium From Leptin‐Treated BMSCs Promotes MUVEC Tubulogenesis and Differentiation

**FIGURE 4:**
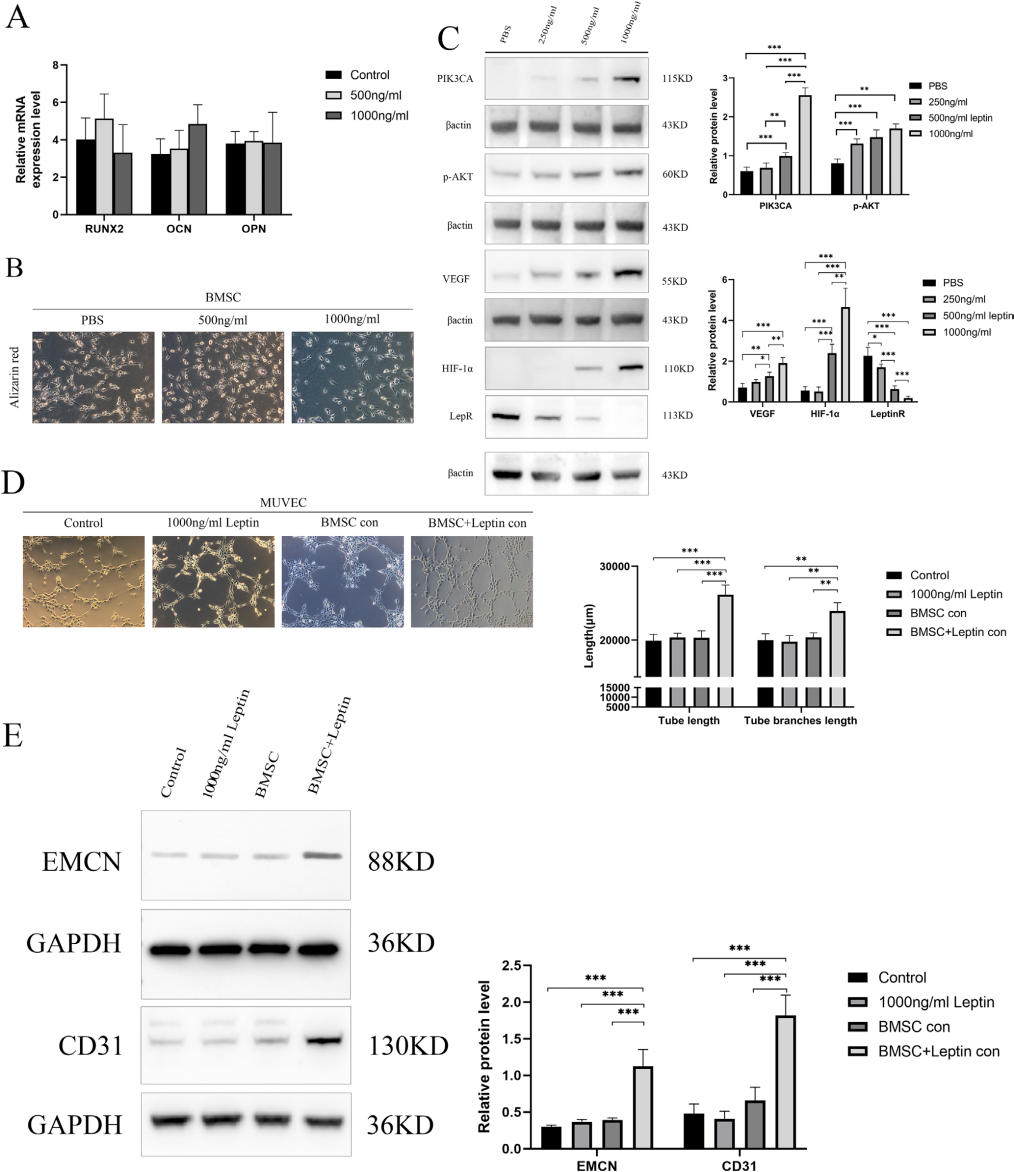
LEP does not directly affect the osteogenic activity of BMSCs or the tubulogenesis ability of MUVECs. However, conditioned medium derived from LEP‐treated BMSCs enhances the tubulogenesis ability of MUVECs and promotes their differentiation towards H‐type vessels. (A) Relative expression levels of osteogenic marker genes (RUNX2, OCN, OPN) in BMSCs treated with different concentrations of LEP, measured by RT‐PCR (*n* = 5). (B) Alizarin Red S staining results of BMSCs treated with different concentrations of LEP. (C) Western blot analysis of PIK3CA, p‐AKT, VEGF, HIF‐1α, and LEPR protein levels in BMSCs treated with increasing concentrations of LEP (*n* = 5). (D) Matrigel tubulogenesis assay of MUVECs treated with different conditioned media (as described in the main text). Top panels show representative microscope images; bottom panels show node‐marking diagrams generated by ImageJ software analysis. Total tube length and total branch length were quantified (*n* = 3). (E) Western blot analysis of EMCN and CD31 expression levels in MUVECs subjected to the different treatments described above (*n* = 5). All data are presented as mean ± SEM. *p < 0.05, **p < 0.01, ***p < 0.001.

To investigate the underlying mechanism, we conducted in vitro cell experiments. Mouse bone marrow mesenchymal stem cells (BMSCs) were treated with recombinant leptin at concentrations of 0, 500 ng/mL, and 1000 ng/mL. Subsequent qPCR analysis of extracted total RNA showed no significant differences in the expression of osteogenesis‐related genes (*RUNX2*, *OCN*, *OPN*) across the treatment groups (Figure 4A). Alizarin Red S staining of BMSCs treated with these leptin concentrations revealed no obvious osteogenic differentiation or calcium deposition (Figure 4B).

Western blot analysis was performed on total protein extracted from BMSCs treated with 0, 250 ng/mL, 500 ng/mL, and 1000 ng/mL leptin. The results demonstrated that BMSCs treated with higher leptin concentrations exhibited significantly increased levels of PIK3CA, phosphorylated AKT (p‐AKT), VEGF, and HIF‐1α proteins. Conversely, leptin receptor (LEPR) expression was decreased (Figure 4C).

To assess the paracrine effect, MUVEC cells were treated for 24 h with one of four conditioned media: (1) PBS (control), (2) 1000 ng/mL leptin directly, (3) serum‐free conditioned medium from untreated BMSCs (BMSC con), or (4) serum‐free conditioned medium from BMSCs pre‐treated with 1000 ng/mL leptin (BMSC+Leptin con). A subsequent Matrigel tubulogenesis assay revealed that MUVECs in the BMSC+Leptin con group exhibited a significantly enhanced ability to form tube‐like structures compared to the other three groups (Figure 4D). Western blot analysis of total protein extracted from these MUVECs showed markedly increased expression of the H‐type vessel markers EMCN and CD31 in the BMSC+Leptin con group compared to the others (Figure 4E).

In summary, leptin does not directly induce osteogenic differentiation in BMSCs or enhance the tubulogenic potential of MUVECs. However, it activates the PI3K/AKT signalling pathway in BMSCs, leading to increased secretion of pro‐angiogenic factors. The conditioned medium from these leptin‐stimulated BMSCs potently promotes tubulogenesis and the expression of H‐type vessel‐specific markers in MUVECs.

### 3.5 Inhibition of the PI3K‐AKT Pathway Blocks LEP‐Induced Production of Pro‐Angiogenic Factors in BMSCs and Attenuates Its Promotive Effects on MUVEC Tubulogenesis and Differentiation

Based on the preceding experimental results, we hypothesized that LEP activates the PI3K‐AKT pathway in BMSCs, thereby increasing the expression of pro‐angiogenic and differentiation cytokines such as VEGF and HIF‐1α. This, in turn, promotes the tubulogenesis and differentiation of MUVECs via a paracrine mechanism mediated by the conditioned medium. To validate this hypothesis, BMSC cells were divided into three experimental groups. The first group was treated with 1000 ng/mL Leptin (BMSCs+Leptin group). The second group was treated with 1000 ng/mL Leptin plus 20 μmol/L of the PI3K pathway inhibitor Ly294002 (BMSCs+Leptin+Ly294002 group). The third group received a sham treatment with PBS (Control group).

**FIGURE 5:**
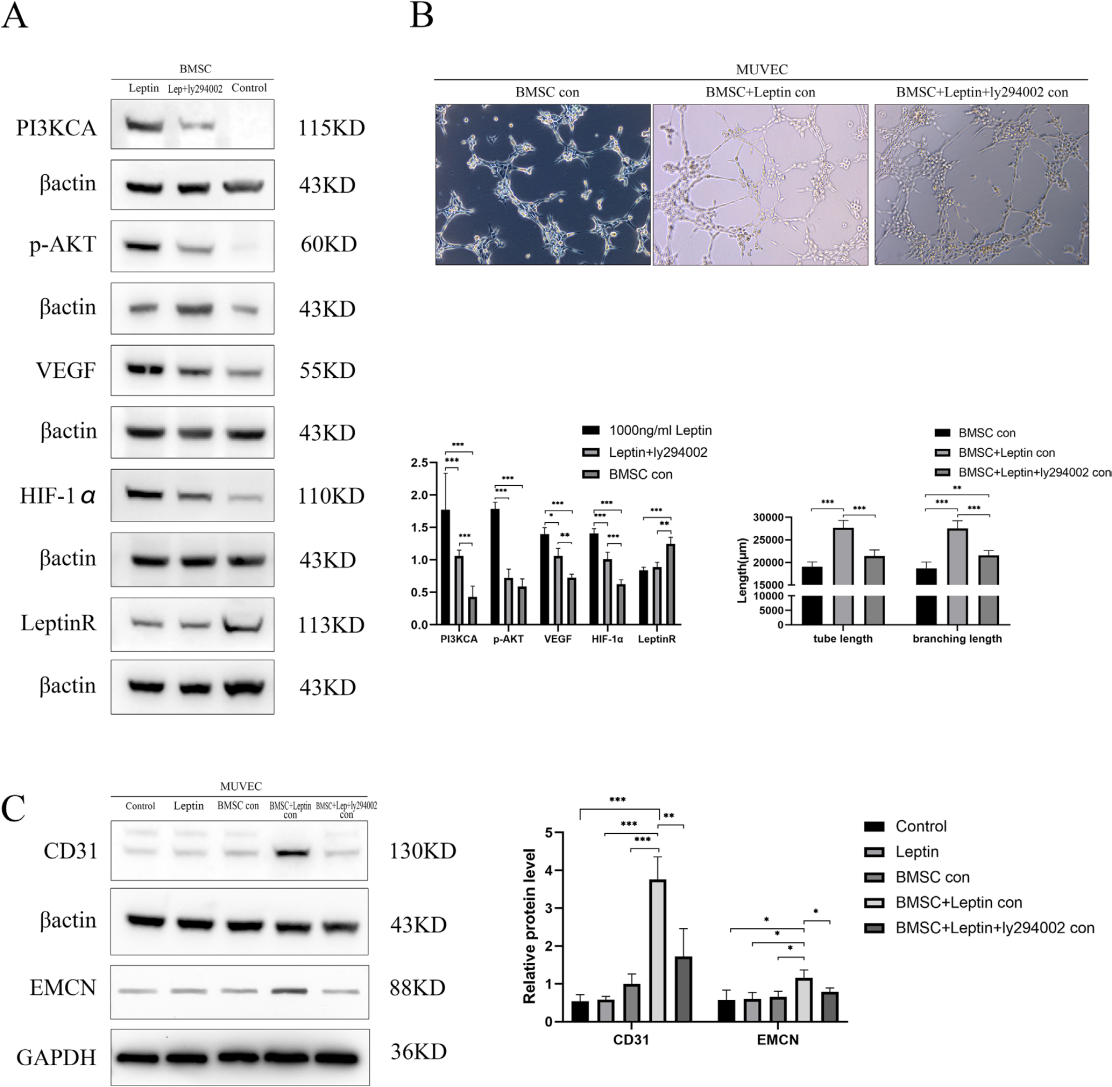
Inhibition of the PI3K‐AKT pathway suppresses LEP‐induced production of pro‐angiogenic factors in BMSCs and its subsequent promotion of MUVEC tubulogenesis and differentiation. (A) Western blot analysis of PI3KCA, p‐AKT, VEGF, HIF‐1α, and LeptinR protein expression levels in total protein extracted from BMSCs subjected to different treatments (as detailed in the main text) (*n* = 3). (B) Comparison of tubulogenesis ability in MUVECs treated with conditioned media from the different BMSC treatment groups. Top panels show original microscope images; bottom panels show node‐marking diagrams generated using ImageJ software analysis (*n* = 3). (C) Western blot analysis of CD31 and EMCN levels in total protein extracted from MUVEC cells subjected to different treatments (for details, see the main text) (*n* = 3). All data are presented as mean ± SEM. *p < 0.05, **p < 0.01, ***p < 0.001.

Western blot analysis of total protein extracted from these three groups of BMSCs revealed that Leptin treatment increased the levels of PI3KCA, p‐AKT, VEGF, and HIF‐1α proteins, while decreasing LeptinR expression. However, when Ly294002 was co‐administered, the increasing trends in PI3KCA, p‐AKT, VEGF, and HIF‐1α expression were suppressed. Notably, the decreasing trend in LeptinR expression was not affected by the inhibitor (Figure 5A).

Subsequently, MUVEC cells were treated with the serum‐free conditioned media obtained from the aforementioned three BMSC treatment groups. A tubulogenesis assay performed with these MUVECs demonstrated that cells treated with conditioned medium from the first group (BMSCs+Leptin con group) exhibited a significantly enhanced ability to form tubes. In contrast, MUVECs treated with conditioned medium from the inhibitor group (BMSCs+Leptin+Ly294002 con group) showed a restoration of tubulogenesis ability to a level comparable to the untreated control group (BMSCs con) (Figure 5B).

Western blot analysis of total protein extracted from these MUVEC cells confirmed that the group treated with BMSCs+Leptin con conditioned medium had markedly elevated expression levels of CD31 and EMCN compared to the other groups. No statistically significant differences were observed among the other groups. As an additional control, direct treatment of MUVEC cells with 1000 ng/mL leptin (Leptin group) or PBS (Control group) did not result in significant changes in CD31 and EMCN expression levels, which remained consistent with the levels observed in the other non‐BMSCs+Leptin con groups (Figure 5C).

These experimental findings collectively demonstrate that LEP activates the PI3K‐AKT pathway in BMSCs, leading to increased expression of VEGF and HIF‐1α. This, in turn, promotes the tubulogenesis ability and upregulates the expression of EMCN and CD31 in MUVECs. Critically, the inhibitor Ly294002 effectively blocks this entire process by inhibiting the PI3K‐AKT pathway.

### 3.6 Pharmacological Blockade of the PI3K‐AKT Pathway Reduces H‐Type Vessel Formation and Alleviates OA Severity in Obese Mice

Subsequently, we investigated whether pharmacological inhibition of the PI3K‐AKT pathway in obese mice could reduce H‐type vessel formation and mitigate OA severity. Mice were divided into three groups. Group 1 consisted of 5 male leptin‐knockout C57BLKS‐Lep^ob^/J mice (ob/ob). Groups 2 and 3 each consisted of 5 male wild‐type C57BLKS/J mice (BKS). At 8 weeks of age, DMM surgery was performed on the right knee of all mice to induce OA, while the left knee underwent a sham operation as a control. Postoperatively, all three groups were fed a high‐fat diet. Starting from the surgery, Group 3 received a daily oral gavage of ZSTK474 at 400 mg/kg/day (BKS + ZSTK474 group), while the other two groups received 1–2 mL of purified water as a vehicle control. All mice underwent daily running wheel exercise (10 m/min, 60 min), and body weight was measured weekly. At 17 weeks, all mice were euthanized. Blood was collected for serum leptin measurement by ELISA, and knee joint specimens were harvested for micro‐CT analysis. Subsequently, joints were fixed, decalcified, sectioned, and subjected to Safranin O/Fast Green staining, immunohistochemistry, and immunofluorescence staining.

**FIGURE 6:**
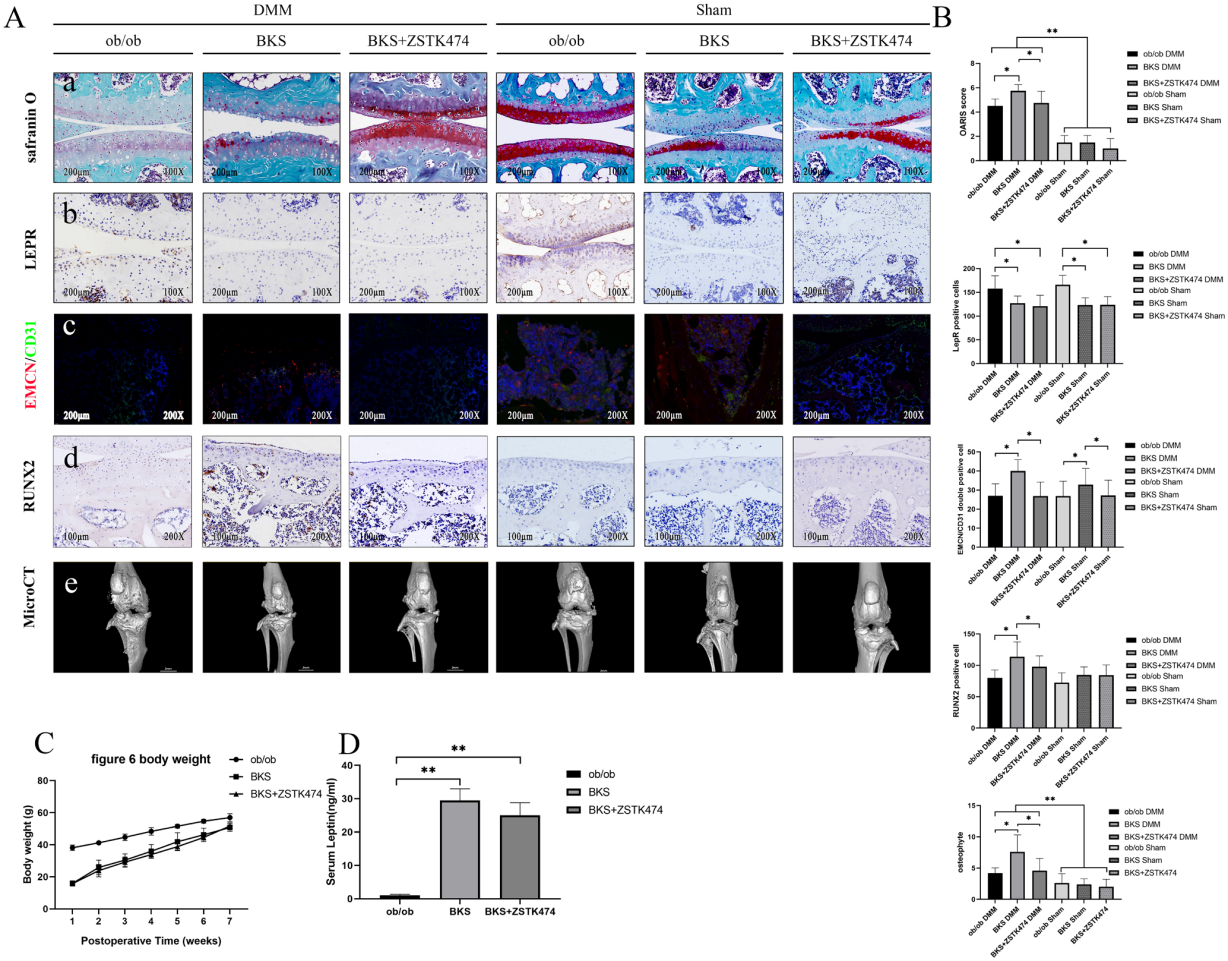
Pharmacological inhibition of the PI3K‐AKT pathway alleviates OA manifestations induced by high serum leptin and reduces H‐type vessel numbers in mice. No osteoarthritic pathological changes were observed in the sham‐operated group (A) Histological analysis of knee joints from C57BLKS/J mice subjected to different treatments. ob/ob: Leptin‐knockout mice; BKS: Wild‐type mice; BKS + ZSTK474: Wild‐type mice receiving daily oral gavage of ZSTK474 (400 mg/kg/day). (a) Safranin O/Fast Green staining; (b) LeptinR immunohistochemical staining; (c) CD31/EMCN immunofluorescence double staining; (d) RUNX2 immunohistochemical staining; (e) Osteophyte quantification by Micro‐CT.(B) Quantitative analysis results of the histological examinations mentioned above (*n* = 5). (C) Body weight change trends of mice in different experimental groups. (D) Plasma leptin concentration in different experimental groups at Week 15. All data are presented as mean ± SEM. *p < 0.05, **p < 0.01, ***p < 0.001.

The body weights of the three groups were comparable at the endpoint, showing no significant difference (Figure 6C). As expected, plasma leptin concentrations were significantly elevated in both the BKS and BKS + ZSTK474 groups compared to the ob/ob group.

As shown in Figure 6A,a, Safranin O/Fast Green staining revealed that the OARSI score was significantly higher in the right knee of the BKS group compared to the other two groups, while no significant difference was observed between the ob/ob and BKS + ZSTK474 groups. Immunohistochemical staining results indicated that the number of LepR‐positive cells was significantly greater in the ob/ob group than in the BKS and BKS + ZSTK474 groups (Figure 6A,b). Concurrently, the number of RUNX2‐positive cells was significantly higher in the BKS group compared to the other two groups (Figure 6A,d). Immunofluorescence double staining for EMCN and CD31 showed a significantly higher number of double‐positive cells (indicating H‐type vessels) in the subchondral bone of the BKS group compared to the other two groups (Figure 6A,c). Micro‐CT scanning confirmed that the number of osteophytes in the right knee joint was also significantly greater in the BKS group than in the other groups (Figure 6A,e). All sham‐operated knee joints showed no obvious OA pathological changes, and no significant differences were observed in histochemical assays among groups.

In summary, obese mice with high serum leptin levels exhibit increased RUNX2 expression, enhanced H‐type vessel formation in the knee joint, and aggravated OA severity. Administration of the PI3K‐AKT pathway inhibitor ZSTK474, or genetic knockout of LEP gene, effectively blocks this trend, reducing H‐type vessel generation and alleviating OA severity.

Therefore, based on the combined in vitro and in vivo evidence, we conclude that the hyperleptinemic state in obese mice induces H‐type vessel formation in the subchondral bone, promotes subchondral bone remodelling, and consequently exacerbates OA. This process is mediated by LEP through the activation of the PI3K‐AKT pathway. Genetic ablation of LEP or pharmacological inhibition of the PI3K‐AKT pathway can suppress this process, thereby protecting the knee joint.

## 4 Discussion

Most previous research on the relationship between leptin and OA has focused geographically and histologically on the infrapatellar fat pad, synovium, and chondrocytes. Since cartilage is avascular and relies on synovial fluid for nutrition, the infrapatellar fat pad and synovium are considered the main sources of leptin within the joint space [25]. Consistent with this, studies have reported higher leptin expression in the infrapatellar fat pad, synovium, synovial fluid, and chondrocytes of OA patients or animal models compared to healthy controls [26] with expression levels positively correlating with OA severity [27]. In contrast, our study examined knee joint subchondral bone and systemic circulation. Bioinformatic analysis, RT‐PCR, and immunohistochemistry consistently demonstrated reduced expression of Leptin and LEPR in the subchondral bone of OA patients, despite significantly higher serum leptin levels. Elevated serum leptin is a common feature in individuals with obesity and metabolic syndrome [28], who are also at high risk for OA. We postulate that the paradoxical decrease in Leptin and LEPR expression in the subchondral bone, contrary to the trend in intra‐articular tissues, may be a manifestation of leptin resistance. The persistent action of high serum leptin can lead to tissue resistance, impairing leptin's weight‐regulating functions and being directly linked to adipose tissue insulin resistance [29]. One characteristic of leptin resistance is the downregulation of leptin and LEPR expression in target tissues [30]. The well‐vascularized subchondral bone, being directly exposed to high circulating leptin levels, may be more prone to developing leptin resistance compared to the relatively avascular cartilage.

Historically, research on the leptin‐OA relationship has concentrated on inflammatory responses and immune regulation [20, 31]. Structurally similar to cytokines like IL‐6, IL‐11, and IL‐12, leptin can exert pro‐inflammatory effects, mediating inflammatory damage to cartilage [14, 32]. Leptin can also promote macrophage polarization towards the M1 phenotype and their recruitment to inflammatory sites, such as joints, via NF‐κB pathway activation, inducing the secretion of various pro‐inflammatory factors [11, 12, 33]. Our study, however, identified a significant increase in H‐type vessel numbers in the subchondral bone of OA patients. H‐type vessels are markers of active bone metabolism, playing crucial roles in bone development, regeneration, and remodelling, and are implicated in OA pathogenesis. They may promote cartilage degradation by facilitating the invasion of MMP‐9 and inflammatory factors into the cartilage and contribute to osteophyte formation and altering stress distribution through subchondral bone sclerosis [34, 35]. This led us to investigate whether leptin might exacerbate OA by promoting H‐type vessel genesis.

Our animal experiments confirmed that high‐fat‐diet‐fed (HFD) C57 mice developed obesity and exhibited significantly elevated serum leptin levels. Compared to leptin‐knockout mice under the same dietary conditions, these wild‐type mice had more severe OA pathology, more osteophytes, increased RUNX2 expression, decreased LepR expression, and a greater abundance of H‐type vessels in the subchondral bone. Given the comparable body weights between the groups, the influence of differential mechanical loading could be excluded, suggesting that serum leptin indeed promotes H‐type vessel formation and exacerbates OA. Several studies on tumour angiogenesis have shown that leptin activates the PI3K‐AKT pathway, inducing the expression of VEGF and HIF‐1α, thereby promoting angiogenesis and H‐type vessel differentiation [36, 37, 38, 39]. Our cell experiments demonstrated that while leptin did not directly induce osteogenic differentiation in BMSCs, it activated the PI3K‐AKT pathway and increased VEGF and HIF‐1α expression. Conditioned medium from these leptin‐treated BMSCs significantly enhanced the tubulogenesis ability of MUVECs and upregulated EMCN and CD31 expression, whereas direct leptin treatment on MUVECs had no such effect. The addition of the PI3K pathway inhibitor Ly294002 alongside leptin blocked the activation of the PI3K pathway and the subsequent pro‐angiogenic effects. Correspondingly. In mice, oral administration of the PI3K‐AKT inhibitor ZSTK474 to HFD fed C57 mice with high serum leptin significantly reduced H‐type vessel numbers and RUNX2 expression, resulting in less severe OA and fewer osteophytes compared to untreated mice. This confirms that inhibiting the PI3K‐AKT pathway effectively mitigates serum leptin‐mediated H‐type vessel formation in the subchondral bone and protects the knee joint. Interestingly, this intervention did not appear to affect subchondral bone LEPR expression.

Research on leptin's effects on angiogenesis is relatively limited and predominantly focused on cancer. The chick chorioallantoic membrane assay has shown that leptin can induce VEGF expression via PI3K/AKT/mTOR activation, stimulating new blood vessel formation [38]. Beyond VEGF, PDGFR‐β and HIF‐1α are also recognized as angiogenic factors regulated by the PI3K/AKT pathway, capable of inducing vasculogenesis and vessel ingrowth [40]. Studies on mouse breast cancer cells found that leptin activates the PI3K‐AKT pathway to induce HIF‐1α production, which in turn stimulates VEGF secretion—a process inhibited by the PI3K inhibitor Wortmannin. In an oral squamous cell carcinoma model, leptin was also shown to promote angiogenesis by increasing the expression of HIF‐1α and other angiogenic factors [41]. In the context of bone and joint tissue, research has indicated that reducing TGF‐β secretion from BMSCs can decrease subchondral H‐type vessel formation, mitigating osteophyte generation and vascular invasion into cartilage. Bai et al., in an OA animal model, discovered that mTORC1 pathway activation promotes VEGF‐A secretion, which drives H‐type vessel formation in the subchondral bone; the ensuing nutrient supply further enhances mTORC1 activity, creating a positive feedback loop that accelerates OA progression [42]. That study did not identify the initial trigger for H‐type vessel ingrowth and mTORC1 activation. However, given that leptin is known to activate mTORC1 via the PI3K‐AKT pathway to secrete VEGF and promote angiogenesis, it is plausible that leptin serves as this initiating factor [43]. These findings align with our experimental results: high serum leptin concentrations indeed promote VEGF and HIF‐1α generation by activating the PI3K‐AKT pathway, thereby fostering H‐type vessel formation and exacerbating OA progression. Blocking this pathway inhibits these effects and protects the knee joint. Therefore, targeting leptin may represent a potential therapeutic strategy for OA prevention and treatment.

Naturally, this study has certain limitations. First, to create mice with different serum leptin levels, we used global leptin gene‐knockout mice. Total knockout (TKO) may cause uncontrolled effects on tissues and organs outside the knee joint, potentially confounding the experimental outcomes. However, tissue or organ‐specific conditional knockout might not fully replicate the systemic changes in serum leptin levels, presenting an inherent dilemma in experimental design. Second, while our study, by controlling for serum leptin and employing pathway blockade, preliminarily confirms that leptin likely promotes H‐type vessel hyperplasia via PI3K‐AKT pathway activation to induce OA, the detailed molecular interactions and direct evidence of biomolecular crosstalk remain to be fully elucidated and require further investigation.

## Author Contributions

**Ruifu Li:** investigation, formal analysis, data curation, writing – original draft. **Jun Qin:** investigation, methodology. **Zhao Xi:** investigation, methodology. **Tao Liu:** investigation, methodology. **Shitong Luo:** investigation, methodology. **Jian Zhang:** conceptualization, supervision, project administration, funding acquisition, writing – review and editing.

## Funding

This work was supported by the Chongqing Science and Health Joint Medical Research Project (Grant Number: 2025MSXM087), the Chongqing Science and Technology Commission (Grant Number: CSTB2023NSCQ‐MSX0640), and the Chongqing Municipal Health Commission (Grant Number: 2025MSXM081).

## Ethics Statement

This study strictly adhered to the principles of laboratory animal ethics and welfare. All procedures and research protocols involving experimental animals were approved by the Institutional Review Board of Chongqing Medical University (No. 2021.5.28/LL‐202133). Throughout the study, all animal husbandry and experimental operations complied with the internationally recognized “3R principles” and were continuously implemented under the committee's supervision to ensure ethical standards were met. The use of human tissue samples in this study was approved by the Ethics Committee of University Town Hospital of Chongqing Medical University (ECUH‐CQMU: IIT‐LL‐2025003). As the study utilized existing, de‐identified samples from the University Town Hospital of Chongqing Medical University biobank. All methods were carried out in accordance with the relevant guidelines and regulations.

## Conflicts of Interest

The authors declare no conflicts of interest.

## Data Availability

The bioinformatic dataset (GSE51588) supporting the findings of this study is publicly available in the GEO repository. All other data generated or analysed during this study, including source data for the graphs, are included in this published article or are available from the corresponding author on reasonable request.

## References

[1] S. Safiri , A.‐A. Kolahi , E. Smith , et al., “Global, Regional and National Burden of Osteoarthritis 1990‐2017: A Systematic Analysis of the Global Burden of Disease Study 2017,” Annals of the Rheumatic Diseases 79 (2020): 819–828, 10.1136/annrheumdis-2019-216515. 32398285

[2] R. Gherghel , D.‐A. Iordan , M.‐D. Mocanu , A. Onu , and I. Onu , “Osteoarthritis Is Not a Disease, but Rather an Accumulation of Predisposing Factors. A Systematic Review,” Balneo & PRM Research Journal 12 (2021): 218–226.

[3] M. Blagojevic , C. Jinks , A. Jeffery , and K. P. Jordan , “Risk Factors for Onset of Osteoarthritis of the Knee in Older Adults: A Systematic Review and Meta‐Analysis,” Osteoarthritis and Cartilage 18 (2010): 24–33, 10.1016/j.joca.2009.08.010. 19751691

[4] S. Panunzi , S. Maltese , A. De Gaetano , E. Capristo , S. R. Bornstein , and G. Mingrone , “Comparative Efficacy of Different Weight Loss Treatments on Knee Osteoarthritis: A Network Meta‐Analysis,” Obesity Reviews: An Official Journal of the International Association for the Study of Obesity 22 (2021): e13230, 10.1111/obr.13230. 33855769

[5] G. S. Hotamisligil , “Inflammation and Metabolic Disorders,” Nature 444 (2006): 860–867, 10.1038/nature05485. 17167474

[6] X. Wang , F. Li , C. Fan , C. Wang , and H. Ruan , “Effects and Relationship of ERK1 and ERK2 in Interleukin‐1β‐Induced Alterations in MMP3, MMP13, Type II Collagen and Aggrecan Expression in Human Chondrocytes,” International Journal of Molecular Medicine 27 (2011): 583–589, 10.3892/ijmm.2011.611. 21305249

[7] Y.‐P. Su , C.‐N. Chen , K.‐C. Huang , et al., “Leptin Induces MMP1/13 and ADAMTS 4 Expressions Through Bone Morphogenetic Protein‐2 Autocrine Effect in Human Chondrocytes,” Journal of Cellular Biochemistry 119 (2018): 3716–3724, 10.1002/jcb.26593. 29236309

[8] A. Leonidou , P. Lepetsos , M. Mintzas , et al., “Inducible Nitric Oxide Synthase as a Target for Osteoarthritis Treatment,” Expert Opinion on Therapeutic Targets 22 (2018): 299–318, 10.1080/14728222.2018.1448062. 29504411

[9] R. Wiegertjes , F. A. J. van de Loo , and E. N. Blaney Davidson , “A Roadmap to Target Interleukin‐6 in Osteoarthritis,” Rheumatology (Oxford, England) 59 (2020): 2681–2694, 10.1093/rheumatology/keaa248. 32691066 PMC7516110

[10] M. J. López‐Armada , B. Caramés , M. A. Martín , et al., “Mitochondrial Activity Is Modulated by TNFα and IL‐1β in Normal Human Chondrocyte Cells,” Osteoarthritis and Cartilage 14 (2006): 1011–1022, 10.1016/j.joca.2006.03.008. 16679036

[11] C. Li , M. M. Xu , K. Wang , A. J. Adler , A. T. Vella , and B. Zhou , “Macrophage Polarization and Meta‐Inflammation,” Translational Research 191 (2018): 29–44, 10.1016/j.trsl.2017.10.004. 29154757 PMC5776711

[12] A. R. Sun , X. Wu , B. Liu , et al., “Pro‐Resolving Lipid Mediator Ameliorates Obesity Induced Osteoarthritis by Regulating Synovial Macrophage Polarisation,” Scientific Reports 9 (2019): 426, 10.1038/s41598-018-36909-9. 30674985 PMC6344566

[13] Y. Kamada , T. Takehara , and N. Hayashi , “Adipocytokines and Liver Disease,” Journal of Gastroenterology 43 (2008): 811–822, 10.1007/s00535-008-2213-6. 19012034

[14] A. Koskinen , K. Vuolteenaho , T. Moilanen , and E. Moilanen , “Resistin as a Factor in Osteoarthritis: Synovial Fluid Resistin Concentrations Correlate Positively With Interleukin 6 and Matrix Metalloproteinases MMP‐1 and MMP‐3,” Scandinavian Journal of Rheumatology 43 (2014): 249–253, 10.3109/03009742.2013.853096. 24780007

[15] J. F. Nishimuta and M. E. Levenston , “Meniscus Is More Susceptible Than Cartilage to Catabolic and Anti‐Anabolic Effects of Adipokines,” Osteoarthritis and Cartilage 23 (2015): 1551–1562, 10.1016/j.joca.2015.04.014. 25917638 PMC4558246

[16] M. Gosset , F. Berenbaum , C. Salvat , et al., “Crucial Role of Visfatin/Pre–B Cell Colony‐Enhancing Factor in Matrix Degradation and Prostaglandin E2 Synthesis in Chondrocytes: Possible Influence on Osteoarthritis,” Arthritis & Rheumatism 58 (2008): 1399–1409, 10.1002/art.23431. 18438860

[17] H. Oh , J. S. Kwak , S. Yang , et al., “Reciprocal Regulation by Hypoxia‐Inducible Factor‐2α and the NAMPT‐NAD+‐SIRT Axis in Articular Chondrocytes Is Involved in Osteoarthritis,” Osteoarthritis and Cartilage 23 (2015): 2288–2296, 10.1016/j.joca.2015.07.009. 26209889

[18] O. P. Stannus , Y. Cao , B. Antony , et al., “Cross‐Sectional and Longitudinal Associations Between Circulating Leptin and Knee Cartilage Thickness in Older Adults,” Annals of the Rheumatic Diseases 74 (2015): 82–88, 10.1136/annrheumdis-2013-203308. 24078677

[19] M. Massengale , W. M. Reichmann , E. Losina , D. H. Solomon , and J. N. Katz , “The Relationship Between Hand Osteoarthritis and Serum Leptin Concentration in Participants of the Third National Health and Nutrition Examination Survey,” Arthritis Research & Therapy 14 (2012): R132, 10.1186/ar3864. 22651805 PMC3446514

[20] E. Neumann , S. Junker , G. Schett , K. Frommer , and U. Müller‐Ladner , “Adipokines in Bone Disease,” Nature Reviews Rheumatology 12 (2016): 296–302, 10.1038/nrrheum.2016.49. 27080691

[21] J. Santos‐Alvarez , R. Goberna , and V. Sánchez‐Margalet , “Human Leptin Stimulates Proliferation and Activation of Human Circulating Monocytes,” Cellular Immunology 194 (1999): 6–11, 10.1006/cimm.1999.1490. 10357875

[22] Y.‐C. Han , B. Ma , S. Guo , et al., “Leptin Regulates Disc Cartilage Endplate Degeneration and Ossification Through Activation of the MAPK‐ERK Signalling Pathway In Vivo and In Vitro,” Journal of Cellular and Molecular Medicine 22 (2018): 2098–2109, 10.1111/jcmm.13398. 29372627 PMC5867127

[23] Z. M. Zhang , C. Shen , H. Li , et al., “Leptin Induces the Apoptosis of Chondrocytes in an In Vitro Model of Osteoarthritis via the JAK2‐STAT3 Signaling Pathway,” Molecular Medicine Reports 13 (2016): 3684–3690, 10.3892/mmr.2016.4970. 26936086

[24] Y. Wang , Z. Xu , J. Wang , and S. Xu , “DUSP19, a Downstream Effector of Leptin, Inhibits Chondrocyte Apoptosis via Dephosphorylating JNK During Osteoarthritis Pathogenesis,” Molecular BioSystems 12 (2016): 721–728, 10.1039/C5MB00776C. 26751999

[25] J. Conde , M. Scotece , V. López , et al., “Differential Expression of Adipokines in Infrapatellar Fat Pad (IPFP) and Synovium of Osteoarthritis Patients and Healthy Individuals,” Annals of the Rheumatic Diseases 73 (2014): 631–633, 10.1136/annrheumdis-2013-204189. 24099837

[26] J. H. Ku , C. K. Lee , B. S. Joo , et al., “Correlation of Synovial Fluid Leptin Concentrations With the Severity of Osteoarthritis,” Clinical Rheumatology 28 (2009): 1431–1435. 19662330 10.1007/s10067-009-1242-8

[27] M. Yan , J. Zhang , H. Yang , and Y. Sun , “The Role of Leptin in Osteoarthritis,” Medicine 97 (2018): e0257, 10.1097/md.0000000000010257. 29620639 PMC5902277

[28] J.‐P. Tsai , “The Association of Serum Leptin Levels With Metabolic Diseases,” Tzu Chi Medical Journal 29 (2017): 192–196, 10.4103/tcmj.tcmj_123_17. 29296046 PMC5740690

[29] S.‐E. Kim , J. Choo , J. Yoon , et al., “Genome‐Wide Analysis Identifies Colonic Genes Differentially Associated With Serum Leptin and Insulin Concentrations in C57BL/6J Mice Fed a High‐Fat Diet,” PLoS One 12 (2017): e0171664, 10.1371/journal.pone.0171664. 28170448 PMC5295695

[30] A. M. Madiehe , S. Hebert , T. D. Mitchell , and R. B. S. Harris , “Strain‐Dependent Stimulation of Growth in Leptin‐Treated Obese Db/Db Mice,” Endocrinology 143 (2002): 3875–3883, 10.1210/en.2002-220362. 12239099

[31] V. Abella , M. Scotece , J. Conde , et al., “Leptin in the Interplay of Inflammation, Metabolism and Immune System Disorders,” Nature Reviews Rheumatology 13 (2017): 100–109, 10.1038/nrrheum.2016.209. 28053336

[32] T. Wang and C. He , “Pro‐Inflammatory Cytokines: The Link Between Obesity and Osteoarthritis,” Cytokine & Growth Factor Reviews 44 (2018): 38–50, 10.1016/j.cytogfr.2018.10.002. 30340925

[33] L. Monteiro , J. A. D. S. Pereira , L. Palhinha , and P. M. M. Moraes‐Vieira , “Leptin in the Regulation of the Immunometabolism of Adipose Tissue‐Macrophages,” Journal of Leukocyte Biology 106 (2019): 703–716, 10.1002/JLB.MR1218-478R. 31087711

[34] J. Zhang , J. Pan , and W. Jing , “Motivating Role of Type H Vessels in Bone Regeneration,” Cell Proliferation 53 (2020): e12874, 10.1111/cpr.12874. 33448495 PMC7507571

[35] S. M. Hussain , C. Dawson , Y. Wang , et al., “Vascular Pathology and Osteoarthritis: A Systematic Review,” Journal of Rheumatology 47 (2020): 748–760, 10.3899/jrheum.181236. 31154415

[36] R. R. Gonzalez‐Perez , Y. Xu , S. Guo , A. Watters , W. Zhou , and S. J. Leibovich , “Leptin Upregulates VEGF in Breast Cancer via Canonic and Non‐Canonical Signalling Pathways and NFκB/HIF‐1α Activation,” Cellular Signalling 22 (2010): 1350–1362, 10.1016/j.cellsig.2010.05.003. 20466060 PMC2928711

[37] A. Calgani , S. Delle Monache , P. Cesare , C. Vicentini , M. Bologna , and A. Angelucci , “Leptin Contributes to Long‐Term Stabilization of HIF‐1α in Cancer Cells Subjected to Oxygen Limiting Conditions,” Cancer Letters 376 (2016): 1–9, 10.1016/j.canlet.2016.03.027. 26996298

[38] R. Manjunathan , N. Devarajan , and M. Ragunathan , “Possible Mechanism of Human Recombinant Leptin‐Induced VEGF A Synthesis via PI3K/Akt/mTOR/S6 Kinase Signaling Pathway While Inducing Angiogenesis: An Analysis Using Chicken Chorioallantoic Membrane Model,” Journal of Vascular Research 58 (2021): 343–360, 10.1159/000516498. 34167108

[39] Q. Cui , Y. Zhang , N. Tian , et al., “Leptin Promotes Angiogenesis via Pericyte STAT3 Pathway Upon Intracerebral Hemorrhage,” Cells 11 (2022): 2755. 36078162 10.3390/cells11172755 PMC9454866

[40] H. Wang , Y. Yin , W. Li , et al., “Over‐Expression of PDGFR‐β Promotes PDGF‐Induced Proliferation, Migration, and Angiogenesis of EPCs Through PI3K/Akt Signaling Pathway,” PLoS One 7 (2012): e30503, 10.1371/journal.pone.0030503. 22355314 PMC3280261

[41] E. M. Sobrinho Santos , T. A. Guimarães , H. O. Santos , et al., “Leptin Acts on Neoplastic Behavior and Expression Levels of Genes Related to Hypoxia, Angiogenesis, and Invasiveness in Oral Squamous Cell Carcinoma,” Tumor Biology 39 (2017): 1010428317699130, 10.1177/1010428317699130. 28459203

[42] R. Wang and B. Xu , “TGFβ1‐Modified MSC‐Derived Exosome Attenuates Osteoarthritis by Inhibiting PDGF‐BB Secretion and H‐Type Vessel Activity in the Subchondral Bone,” Acta Histochemica 124 (2022): 151933, 10.1016/j.acthis.2022.151933. 35933783

[43] A. Ray and M. P. Cleary , “The Potential Role of Leptin in Tumor Invasion and Metastasis,” Cytokine & Growth Factor Reviews 38 (2017): 80–97, 10.1016/j.cytogfr.2017.11.002. 29158066 PMC5720178

