# Correction to ‘Serum Leptin Exacerbates Osteoarthritis by Promoting Subchondral Bone H‐Type Vessel Angiogenesis via Activation of the PI3K/AKT Pathway’

**DOI:** 10.1111/jcmm.71376

**Published:** 2026-10-06

R. Li , Z. Xi , S. Luo , J. Qin , T. Liu , and J. Zhang , “Serum Leptin Exacerbates Osteoarthritis by Promoting Subchondral Bone H‐Type Vessel Angiogenesis via Activation of the PI3K/AKT Pathway,” Journal of Cellular and Molecular Medicine 30, no. 13 (2026): e71232, 10.1111/jcmm.71232 PMC1331471542374652

In Figure 6A(c), the representative CD31/EMCN immunofluorescence image for the DMM BKS+ZSTK474 group was inadvertently duplicated from the DMM ob/ob group during figure assembly. The correct representative CD31/EMCN immunofluorescence image for the DMM BKS+ZSTK474 group is shown below.

**FIGURE 6:**
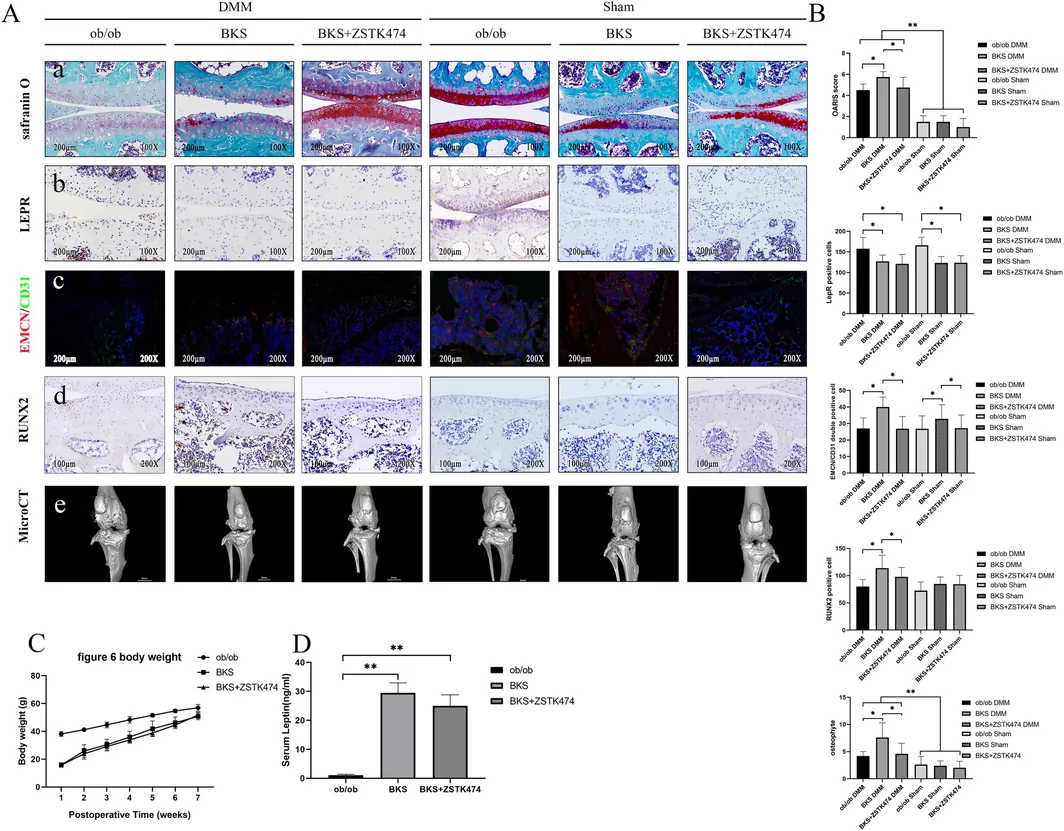
Pharmacological inhibition of the PI3K‐AKT pathway alleviates OA manifestations induced by high serum leptin and reduces H‐type vessel numbers in mice. No osteoarthritic pathological changes were observed in the sham‐operated group (A) Histological analysis of knee joints from C57BLKS/J mice subjected to different treatments. ob/ob: Leptin‐knockout mice; BKS: Wild‐type mice; BKS + ZSTK474: Wild‐type mice receiving daily oral gavage of ZSTK474 (400 mg/kg/day). (a) Safranin O/Fast Green staining; (b) LeptinR immunohistochemical staining; (c) CD31/EMCN immunofluorescence double staining; (d) RUNX2 immunohistochemical staining; (e) Osteophyte quantification by Micro‐CT. (B) Quantitative analysis results of the histological examinations mentioned above (*n* = 5). (C) Body weight change trends of mice in different experimental groups. (D) Plasma leptin concentration in different experimental groups at Week 15. All data are presented as mean ± SEM. *p < 0.05, **p < 0.01, ***p < 0.001.

We apologize for this error.

